# Memetic Cuckoo-Search-Based Optimization in Machining Galvanized Iron

**DOI:** 10.3390/ma13143047

**Published:** 2020-07-08

**Authors:** Kanak Kalita, Ranjan Kumar Ghadai, Lenka Cepova, Ishwer Shivakoti, Akash Kumar Bhoi

**Affiliations:** 1 Department of Mechanical Engineering, Vel Tech Rangarajan Dr Sagunthala R&D Institute of Science and Technology, Avadi 600 062, India; 2 Department of Mechanical Engineering, Sikkim Manipal Institute of Technology, Sikkim Manipal University, Majhitar 737 136, India; ranjan.g@smit.smu.edu.in (R.K.G.); ishwar.siwa@gmail.com (I.S.); 3 VSB-TU Ostrava, Faculty of Mechanical Engineering, 17. listopadu 2172/15, 708 00 Ostrava, Czech Republic; lenka.cepova@vsb.cz; 4 Department of Electrical and Electronics Engineering, Sikkim Manipal Institute of Technology, Sikkim Manipal University, Majhitar 737 136, India; akashkrbhoi@gmail.com

**Keywords:** regression analysis, material removal rate (MRR), cuckoo search, optimization

## Abstract

In this article, an improved variant of the cuckoo search (CS) algorithm named Coevolutionary Host-Parasite (CHP) is used for maximizing the metal removal rate in a turning process. The spindle speed, feed rate and depth of cut are considered as the independent parameters that describe the metal removal rate during the turning operation. A data-driven second-order polynomial regression approach is used for this purpose. The training dataset is designed using an L16 orthogonal array. The CHP algorithm is effective in quickly locating the global optima. Furthermore, CHP is seen to be sufficiently robust in the sense that it is able to identify the optima on independent reruns. The CHP predicted optimal solution presents ±10% deviations in the optimal process parameters, which shows the robustness of the optimal solution.

## 1. Introduction

Turning is one of the essential operations in the lathe. During the turning process, the prominent contributing control variables affecting the process are spindle speed, feed rate, depth of cut, etc. [1]. These process variables must be optimized to obtain maximum productivity and reduce the costs involved. Traditionally, researchers have relied upon the use of one factor at a time (OFAT) analysis to determine the optimum process parameters. However, OFAT techniques are very inefficient because they require a lot of experiments to be carried out. Thus, the advent of regression-based or machine learning-based, predictive models (or metamodels) has been a boon. These compact, reusable and easily deployable metamodels have in general, excellent generalization power which makes them extremely reliable. However, the effectiveness of such metamodels depends on numerous factors including the quality, integrity and size of data, and the nonlinearity in the physical process.

Polynomial regression (PR) and its variants are by far the most widely used metamodels, especially in machining/manufacturing process optimization. This is perhaps because they are easy to implement (as they do not require high-level programming skills or use of complicated mathematical principles), easily quantifiable (due to their fixed form) and readily deployable. Santhanakrishnan et al. [2] built a PR model to study the effect of rake angle, nose radius, cutting speed and feed rate on machining of aluminum. Suresh et al. [3] quantified the material removal rate (MRR) and tool wear rate (TWR) in stainless steels by using PR models. Prabhu and Vinayagam [4] used a full factorial design (FFD) based electric discharge machining experimental dataset to build a PR model. Apart from PR, symbolic regression [5], artificial neural network models [6], adaptive neuro-fuzzy models [7] are often used in process parameter metamodeling.

On the one hand, the metamodels can be used to find the main or interactive effects of the process variables, carrying out sensitivity analysis or uncertainty quantification of the process itself. On the other hand, they must be deployed in conjunction with optimization algorithms to determine the optimum process variables. The optimization algorithms can range from gradient-based algorithms [8,9] to robust topology optimization [10] to metaheuristic algorithms [11,12].

Metaheuristic optimization algorithms are a set of popular optimization algorithms that require no assumptions to be made about the problem to be optimized. Genetic algorithm (GA), particle swarm optimization (PSO), differential evolution (DE), cuckoo search (CS), grey wolf optimization (GWO), etc., are some popular metaheuristics used in process parameter optimization. Kilickap and Huseyinoglu [11] used a GA with PR metamodel to optimize the burr height during drilling operations. Kilickap et al. [12] used a similar PR-GA strategy to minimize the material’s surface roughness. Kalita et al. [13] used both GA and PSO to optimize the laser beam settings in a micro-marking process. In a separate study [14], the delamination in composites due to drilling was also optimized by GA and PSO. In both the studies, it was found that PSO, in general, had faster convergence compared to traditional GA. Nevertheless, since its advent in the 1960s, GA has been successfully deployed for solving almost all types of optimization problems.

Saidi et al. [15] used PR metamodels and the concept of desirability function to maximize the MRR and minimize the SR. They found that the depth of cut has the most impact on the MRR, and the DR is adversely affected by increasing feed rate and insert nose radius. Mia et al. [16] compared the performance of the teaching–learning-based algorithm (TLBO) and bacterial foraging optimization (BFO) to carry out a multiobjective scalar (by using weighted-sum approach) optimization in turning applications. TLBO was found to have a better convergence. Warsi et al. [17] reported a 5% reduction in specific cutting energy and a simultaneous 33% improvement in MRR by using a multiobjective approach in turning of aluminum alloys. Mia and Dhar [18] compared the prediction performance of PR and support vector regression (SVR) metamodels in turning of AISI 1060 steel and found that SVR has superior estimation capability. The SVR and PR metamodels were optimized by using GA, and it was reported that a low feed rate, low material hardness and high cutting speed would produce good quality surfaces with less roughness. Laouissi et al. [19] carried out a similar comparison between PR and ANN metamodels while turning gray cast iron.

Though sufficient work has been done to decide the best parameters in the turning procedure, the scope for further research is adequate. Thus, this research attempts to select the ideal combination for maximizing the MRR during turning of galvanized iron. A novel cuckoo search variant called the coevolutionary host-parasite (CHP) optimization algorithm is used in this study. To the best of authors’ knowledge, this is the first attempt to use a cuckoo search or its variant in the optimization of the turning process.

The rest of the paper is organized as follows. A brief explanation of the approaches such as regression analysis, coevolutionary host-parasite (CHP), material and experimental work is included in Section 2. The statistical analysis of the experimental data, metamodeling and CHP-based process optimization is presented in Section 3. Specific inferences established in the study are strained in the last part of the paper.

## 2. Materials and Methods

### 2.1. Experimental Details

The experimental data are taken from Das et al. [1]. The process variables for at levels are indicated in Table 1. In the present work, high-speed steel has been used as a cutting tool. The tool signature is very important for a single-point cutting tool. The tool signature considered in the order of side rake angle, back rake angle, side cutting edge angle, end relief angle, side relief angle, end cutting edge angle and nose radius were 140°, 80°, 190°, 60°, 80°, 80° and 1 mm, respectively.

Galvanized iron was considered as work material for experimentation. MRR was calculated for each set of combinations. Each experiment was repeated thrice to minimize any experimental and human error. The experimentation chart, along with the measured average MRR based on the L16 orthogonal array, is shown in Table 2.

**Figure 1:**
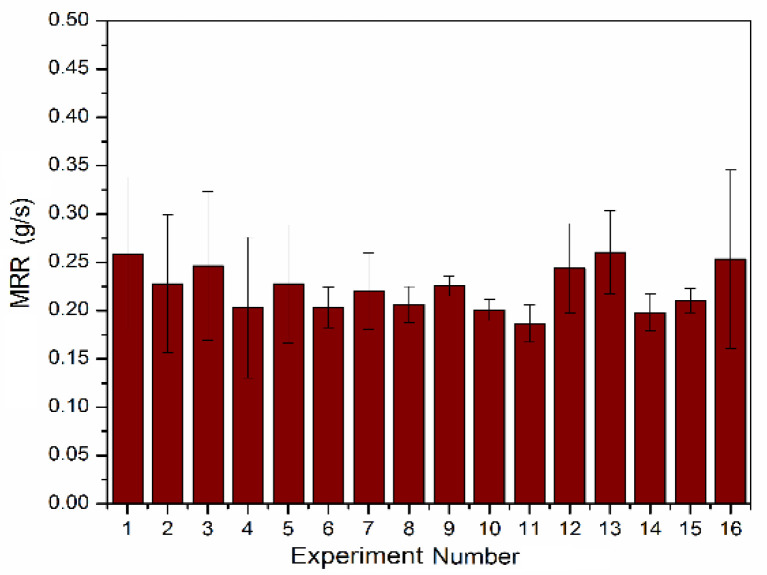
Bar graph of measured average material removal rate (MRR) for each experiment. Error bars show the standard deviation value.

Figure 1 shows the calculated average MRR along with the standard deviation in readings. The MRR can be calculated as per the relation is given below:

(1) $$MRR=\frac{Initial\;weight-Final\;Weight}{Time\;Taken}$$

### 2.2. Regression Analysis

Regression analysis is used for modeling of input and output variables and also can be utilized for predicting the value with the given data set. Interpolation and extrapolation of the data can be analyzed with the help of regression analysis. If the predicted values are within the given range, then it is said to be as interpolation otherwise extrapolation. The relationship between the input and output variable can be obtained with the correlation coefficient R. The fitting of the model is determined by determining the value of *R*^2^ [20,21]. *R*^2^ depicts the proportion of variability for the set of given data accounted by the statistical model. It is given as,

(2) $$R^{2}=1-\frac{SS_{res}}{SS_{tot}}$$

where $SS_{res}$ is the residual sum of squares and $SS_{tot}$ is the total amount of squares which is proportional to the variance of the dataset.

For linear regression, *R*^2^ is just the square of *R*, and the closer *R*^2^ is to 1, the better the model. The sum of the squares of the residuals is the sum of the squares of the *Y* values minus the *Y* values of the model. Second-order models in the polynomial regression are described as,

(3) $$Y=\beta_{0}+\overset{n}{\underset{j=1}{\sum }}\beta_{j}\;x_{j\;}+\overset{n}{\underset{j=1}{\sum }}\beta_{jj}x_{j}^{2}+\overset{n-1}{\underset{i=1}{\sum }}\overset{n}{\underset{j=1}{\sum }}\beta_{ij}\;x_{i\;}x_{j\;}+\varepsilon$$

Here, *Y* is the dependent variable or the response $x_{1\;},x_{2\;},\ldots ,x_{n}$ are the independent variables. $\beta_{0}$,$\;\beta_{1},\;$…, $\beta_{n}$ and $\beta_{j},\beta_{jj\ldots .}$ are the calculated coefficients and the ε is the experimental error. These models can account for quadratic nature in the data variations. If there are any further curvatures in the model, it can be considered by taking higher-order polynomials. In the current study, regression analysis is carried out by using the author-compiled Fortran code.

### 2.3. Optimization Using Improved Cuckoo Search

The cuckoo search algorithm (CS) [22] is a relatively new metaheuristic approach based on the real-life parasitic egg-laying behavior of cuckoos in crow nests. In the current research, the traditional variant of the CS algorithm is robustified by incorporating certain memetic features suggested by Mishra et al. [23]. The improved cuckoo search called as the coevolutionary host-parasite algorithm (CHP) differs from CS in two main ways. On the one hand, it allows the crows to regenerate their nests, thereby allowing coevolution. On the other hand, the probability of detection is dynamic, i.e., as time progresses, if cuckoo and crow interaction is more, the detection rate would also increase.

Initially in the CHP algorithm, a random population of “*n_h_*” hosts and “*n_p_*” parasites are created, and their fitness is measured. For this research, the number of initial host and parasite population is selected as 50 each as the recommendations of Kalita et al. [24]. Each *m*^th^ parasite tries to update its position at the (t+1)^th^ iteration by taking a Lévy flight,

(4) $$p_{t+1}{}^{m}=p_{t}{}^{m}+\left[\alpha \left(r_{1}-0.5\right)\cdot Levy\left(\beta \right)\right]\cdot \left[h_{t}-p_{t}{}^{m}\right]$$

where $\alpha =0.0001+r_{2}{}^{2};\;\beta =\frac{3}{2},\;f\left(h_{t}{}^{k}\right)\;\mathrm{and}\;f\left(p_{t}{}^{m}\right)$ are the fitness of *k*^th^ host and *m*^th^ parasite at the *t*^th^ iteration.

If *m*^th^ parasite’s fitness after-flight is better than its previous fitness, it lays an egg at random so far uninvaded host nest. However, it remains at its old position if its preflight position is superior to the postflight position. In the CHP algorithm, unlike the CS algorithm, the egg may be detected and destroyed by the host. $P_{t}{}^{det}$ i.e., the probability of egg detection is dynamic and following an exponential Gompertz growth curve

(5) $$P_{t+1}{}^{det}=P_{max.}{}^{det}\cdot e^{-2\cdot e^{-{\left(1+\ln\left(1+P_{t}{}^{det}\right)\right)}^{-1}}}$$

In this study, the maximum detection probability, $P_{max.}{}^{det}$ is set at 70%. Since the proposed optimization algorithm draws its inspiration from nature, the maximum 70% rejection criteria are set to mimic the real-life situation as per the study of Bartol et al. [25] on a great reed warbler *Acrocephalus arundinaceus* population, which was heavily parasitized by the common cuckoo *Cuculus canorus*. They found that as much as 73.8% of the artificial cuckoo eggs were rejected.

The egg, if not destroyed, would hatch and the overall *n_p_* best parasites form the next generation parasite population.

The hosts also take a Lévy flight to update their position,

(6) $$h_{t+1}{}^{k}=h_{t}{}^{k}+\left[\omega \left(r_{3}-0.5\right)\cdot Levy\left(\gamma \right)\right]\cdot \left[p_{t}-h_{t}{}^{k}\right]$$

where $\omega =0.0001+r_{4}{}^{2};\;\gamma =\frac{5}{3}$.

Similar to the parasite, the hosts too update their position if their postflight fitness is superior to their preflight position.

An author-compiled Fortran code is used in the current research to carry out the memetic cuckoo-search-based optimization.

## 3. Results and Discussion

### 3.1. Building the Regression Model

The L16 orthogonal array was utilized for designing an experiment and formulating a mathematical relationship to describe the MRR in turning of galvanized iron approximately. Table 3 presents a model statistics summary for MRR. Table 3 depicts that the linear model is not suitable to define the problem appropriately.

As the order increases, significant improvement was observed in *R*^2^ value. Further, if the linear model is switched by the quadratic model, the improvement in *R*^2^ was observed from 5.67% to 84.87%. *R*^2^ depicts how well the regression model is able to explain the variability in the data. For example, if it is 100%, it indicates that the regression model is able to explain all the variability in the data. However, if it is 0%, it indicates that the regression model is no better than the mean or average of the dataset in explaining the variance in the data. However, sometimes the *R*^2^ value can be misleading, as adding more terms to a polynomial regression model will in general increase the *R*^2^. Hence, as emphasized by Kalita et al. [26] in a recent study, all researchers should rely more on the adjusted *R*^2^ values.

### 3.2. ANOVA of the Regression Model

The ANOVA (analysis of variance) for the quadratic model is presented in Table 4. The sum of squares represents the sum of the squared nonconformities from the mean. In an ANOVA, the total sum of squares expresses the total difference that can be ascribed to various factors. The variance related to a specific term is the mean square of the term. It is calculated by dividing the sum of squares by the Degrees of Freedom (df). The model F-value with 3.7404 implies the model is substantial. “Prob > F” higher than 0.05 represents that the model is not significant [27,28]. In such a situation, *N*, *fd*, *N*^2^ and *f*^2^ are the most important model terms. Since there are statistically insignificant terms in the present model, a reduction of the regression model is essential. However, the hierarchy terms should be preserved irrespective of their significance in the model. For example, since the term *fd* is significant for the model, the main effect terms *f* and *d* must be preserved.

The ANOVA for the reduced quadratic model is depicted in Table 4. A small decrease in *R*^2^ is realized when associated with the prior model. As mentioned already associating only *R*^2^ for accepting or rejecting a model can be uncertain. Indeed, a nearer look at both the tables tells that the second model executes well as it has considerably greater adjusted *R*^2^. The F-value of 5.66 designates that the model is significant, and there is only a 1.3% chance that F-value this large could occur due to noise. Hence, the second model is recognized, and the formulation of the MRR equation has been done and is depicted in Equation (7).

(7) $$MRR=0.2682998-0.0009017\;N-0.0907329\;f+0.1697160\;d+0.0003097\;Nf\;-0.3248981\;fd+0.0000013\;N^{2}+0.2660621\;f^{2}$$

### 3.3. Analyzing the Regression Model

**Figure 2:**
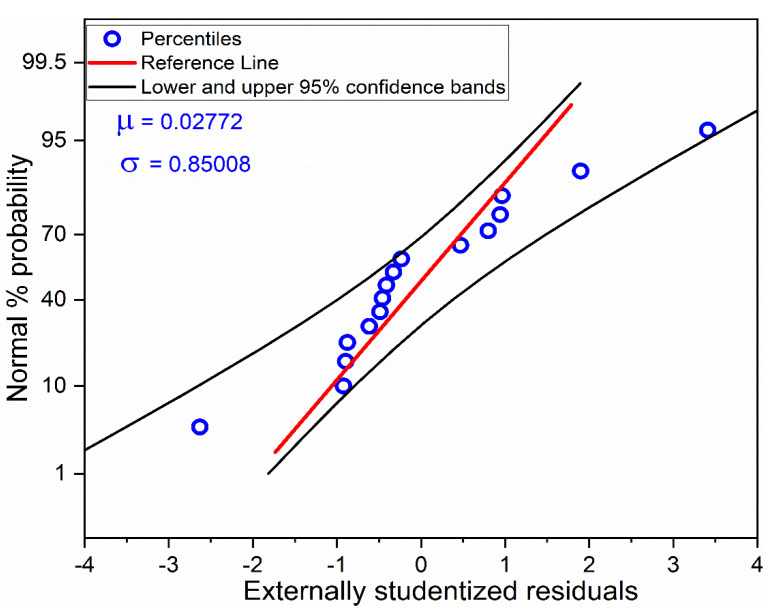
Normal probability plot of residuals for MRR.

The residuals in fitting the experimental MRR values to the polynomial regression model is analyzed using a normal probability plot. The closer the data points are to the theoretical normal probability line, the closer is the distribution of the externally studentized residuals to normal distribution. It is seen from Figure 2 that all the data points are close to the theoretical normal probability line and within the 95% confidence bands which indicates that no possible outlier is present. Further, the lack of any data point clusters in Figure 2 indicates that any data ties are absent. This is also indicative that the measuring resolution is appropriate.

**Figure 3:**
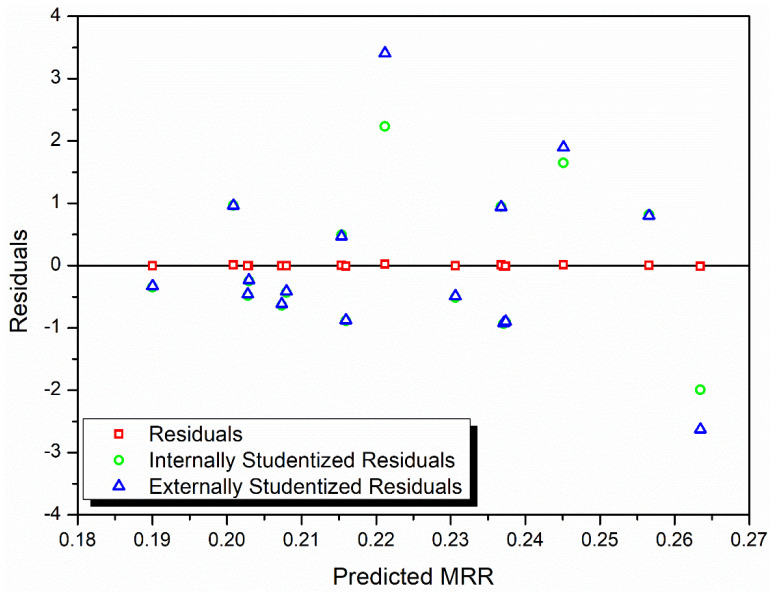
Residuals versus predicted response for MRR.

In Figure 3, the residuals, internally studentized residuals, and externally studentized residuals are plotted against the predicted response values. It is observed that all the data points show a random scatter, which could mean that the hypothesis that constant variance is maintained is not violated [27].

**Figure 4:**
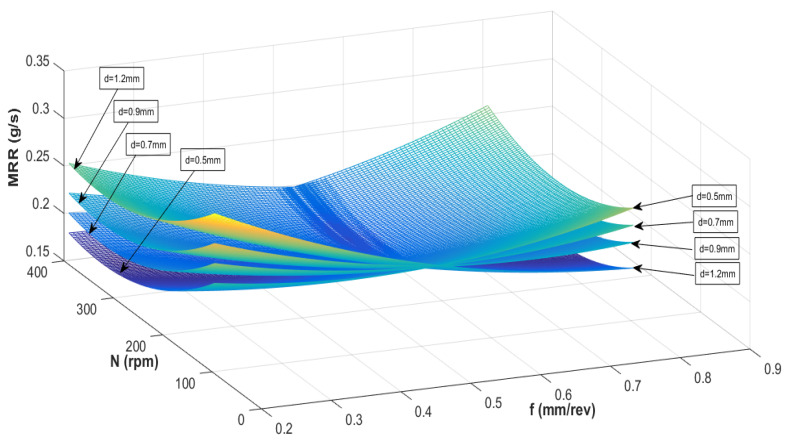
Interaction effect of spindle speed (N) and feed rate (f) on MRR at different depth of cuts.

**Figure 5:**
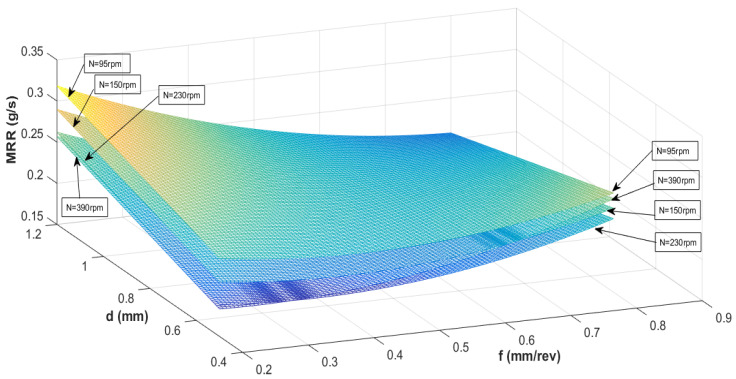
Interaction effect of depth of cut (d) and feed rate (f) on MRR at different spindles.

Figure 4 and Figure 5 show the interaction influence of parameters on the material removal rate. At a small depth of cut, MRR is maximum when feed rate and spindle speed is low. It is seen that the MRR is adversely affected when the feed rate is lowered, and the spindle speed is kept at a maximum. In general, the MRR is higher at any given spindle speed or feed rate for a small depth of cut as compared to a more significant depth of cut. By keeping a low spindle speed and low feed rate, the MRR can be maximized at a high depth of cut. At a low depth of cut and low feed rate, the MRR is minimum when the tool rotation is maximum. It is necessary to verify the presented RSM model before optimizing the process variable with the genetic algorithm.

**Figure 6:**
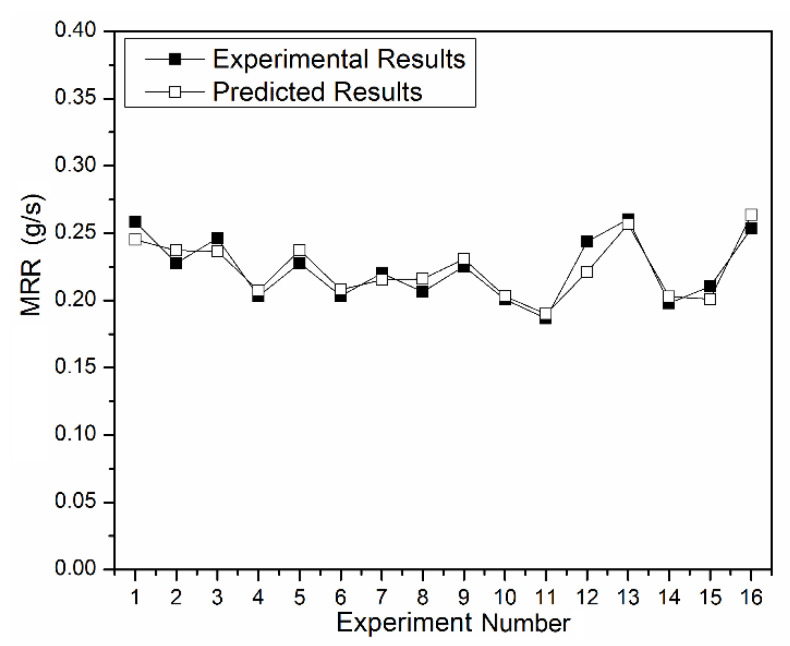
Comparison of the experimental and predicted results.

Figure 6 shows the assessment of the MRR predicted and calculated using Equation (7) with the experimental values. It is observed that the obtained model is sufficient to predict the MRR accurately. The trial number 12 shows the most variation, whereas the others are accurate, and there is only 3.48% of the variation overall.

### 3.4. Process Parameter Optimization with CHP

The polynomial regression equation for MRR obtained in Equation (7) is deployed in conjunction with the Coevolutionary Host-Parasite (CHP) for finding the optimum combination of process parameter values that would maximize the MRR. The optimization problem may be stated as,

Maximize MRR,

with the limits,

95 ≤ *N ≤* 390 0.2 ≤ *f* ≤ 0.8 0.5 *≤ d* ≤ 1.2 (8)

**Figure 7:**
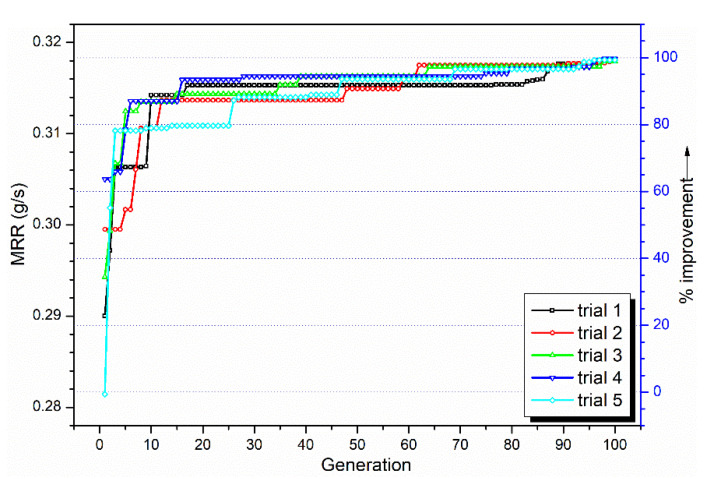
Convergence of Coevolutionary Host-Parasite (CHP) across 100 generations (5 independent trials shown).

The convergence of the CHP algorithm for five typical independent trials is shown in Figure 7. It is seen that in all the trials the CHP algorithm is able to improve its solution state by about 80% (with respect to a predefined baseline value) by the end of the first 20 generations. This shows that the CHP algorithm has rapid improvement capability, and it is able to quickly locate the optimal global zone.

**Figure 8:**
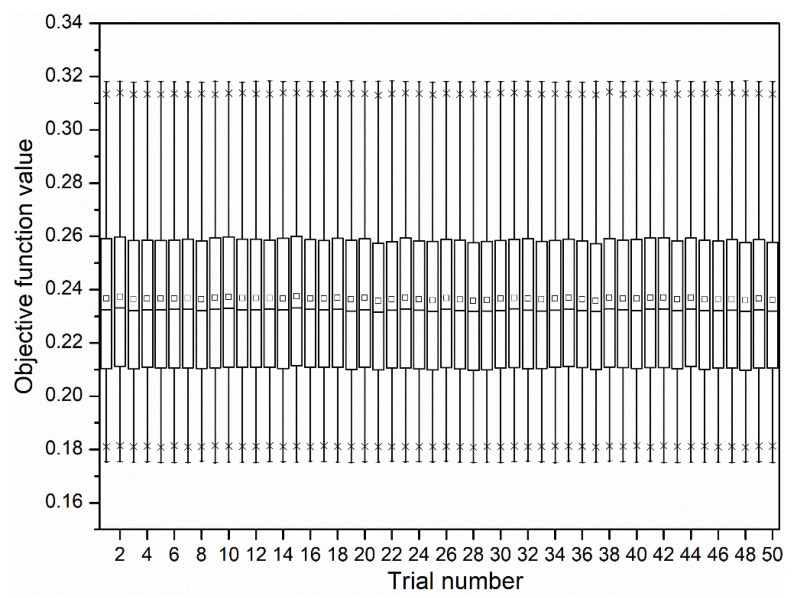
Comparison of total function evaluations of 50 trials.

The total function evaluations for 50 independent trials of the CHP algorithm are plotted in the form of box plots in Figure 8. It is seen that the CHP algorithm is very robust to small changes in its tuning parameters. A similar performance of CHP is seen even in repeated trials.

**Figure 9:**
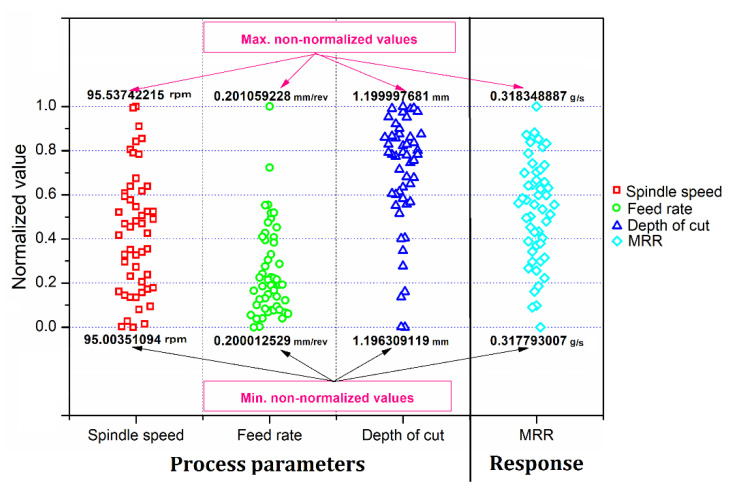
Optimized inputs and output values for 50 independent trials.

The optimized values of the control variables and the dependent variable for 50 independent CHP trials are shown in Figure 9. It is seen that there is negligible variation in the optimized output response obtained in the 50 trials. The difference between the best and the worst solution (output response) among 50 trials is seen to about only a mere 0.15%.

The prediction of optimal control variable combination CHP is listed in Table 5 with the predicted maximum MRR. An experimental confirmation was run to validate the CHP predicted material removal rate. At the optimum parameter combination, the CHP predicted an MRR of 0.318 g/s, whereas the experiments reported it to be 0.326 g/s.

### 3.5. Robustness of CHP Solution

In this section, the robustness of the CHP predicted optimal solution is critically analyzed. Since there is inherent uncertainty associated with traditional machining processes, it is crucial to analyze the robustness of the predicted optimal process parameters. This will provide an understanding of the effect of unwanted human or operational errors that may creep in during the machining process.

**Figure 10:**
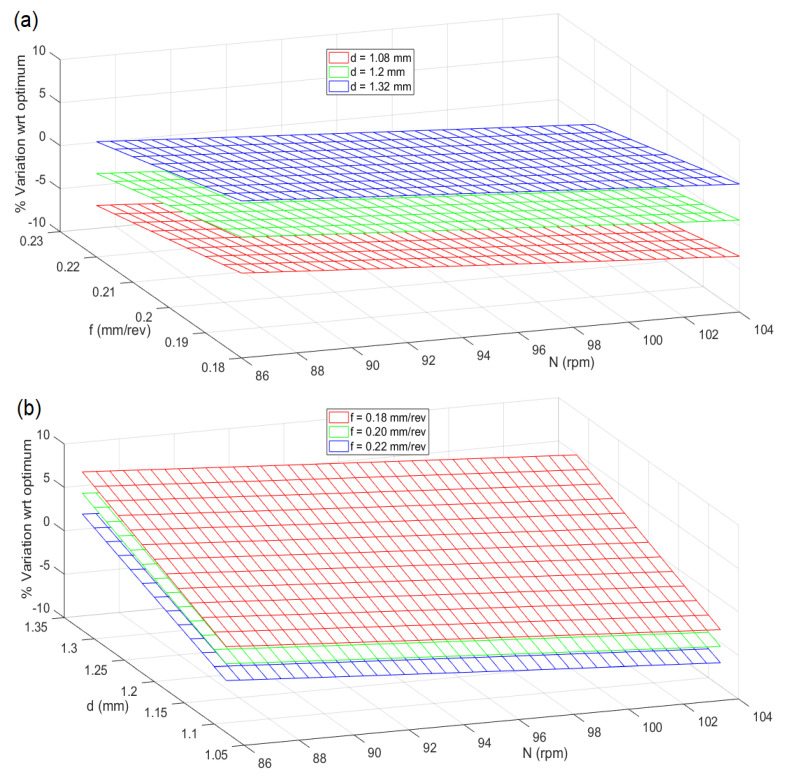

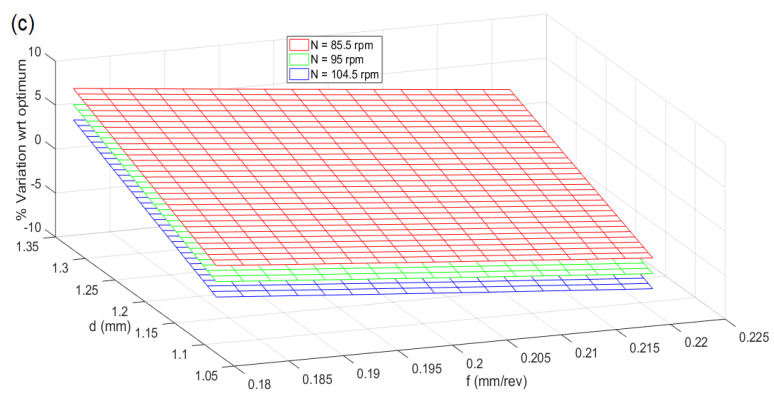
Robustness of the CHP solutions in terms of % variation with respect to optimum when (**a**) spindle speed and feed rate are varied ±10% from their respective optimum values, (**b**) spindle speed and depth of cut are varied ±10% from their individual optimum value (**c**) depth of cut and feed rate are varied ±10% from their respective optimum value.

The percentage variation of the response with respect to optimum at ±10% of the optimum process parameters is plotted in Figure 10. It is seen that the predicted optimal solution is most robust to any changes in spindle speed and feed rate. The optimal solution is likely to be most affected by changes in depth of cut. However, in these cases, the variation in the response concerning the optimal is seen to be within ±7%.

## 4. Conclusions

The effects of machining variables, namely feed rate (F), spindle speed (N), depth of cut (d) on metal removal rate (MRR) have been analyzed during turning galvanized iron using regression-based second-order mathematical models. Based on the experimentation and statistical analysis of the regression model, it is seen that for attaining maximum MRR, the turning tool should be operated at a low feed rate and low spindle speed, if the depth of cut is high. Similarly, the MRR is minimum when the tool is operated at high spindle speed coupled with a low feed rate for low depth of cut. The confirmation experimental trials run on the optimum parameter setting presented that the CHP was very accurate while predicting the global optima. Only 2.45% variation was seen in the CHP predicted optimal and the corresponding experimental output. By carrying out extensive repeated trials of process parameter optimization as well as a comprehensive robustness test, it was shown that the CHP algorithm is very reliable and efficient in locating the global maxima. Thus, the CHP algorithm may be used in conjunction with regression models for the optimization of various other machining and manufacturing operations as it would lead to significant improvement in productivity. Though the focus of the current study has been to increase the productivity of the turning process, the described approach can be easily tuned to tackle other interesting machining problems like improving dimensional accuracy and obtaining better surface finish. Future work of the researchers will focus on some of these issues, as well as tackling the problem from many objective viewpoints, wherein multiple attributes of the machining process can be simultaneously optimized. Further robustification of the machining process parameter optimization approach by coupling the current memetic cuckoo search with advanced machine learning predictive models like neural networks, symbolic regression, support vectors, etc., can be another interesting avenue.

## Figures and Tables

**Table 1:** Parameters and their levels.

Level	Spindle Speed, N (rpm)	Feed Rate, f (mm/rev)	Depth of Cut, d (mm)
1	95	0.2	0.5
2	150	0.45	0.7
3	230	0.5	0.9
4	390	0.8	1.2

**Table 2:** Experimental results [1].

Experiment Number	N (rpm)	f (mm/rev)	d (mm)	Average MRR (g/s)
1	95	0.2	0.5	0.2584
2	95	0.45	0.7	0.2277
3	95	0.5	0.9	0.2463
4	95	0.8	1.2	0.2030
5	150	0.2	0.7	0.2277
6	150	0.45	0.5	0.2033
7	150	0.5	1.2	0.2203
8	150	0.8	0.9	0.2065
9	230	0.2	0.9	0.2253
10	230	0.45	1.2	0.2007
11	230	0.5	0.5	0.1867
12	230	0.8	0.7	0.2437
13	390	0.2	1.2	0.2603
14	390	0.45	0.9	0.1980
15	390	0.5	0.7	0.2103
16	390	0.8	0.5	0.2533

**Table 3:** Performance statistics summary of the various models.

Source	Standard Deviation	*R* ^2^	Adjusted *R*^2^
Linear	0.025	5.67%	−17.91%
2FI	0.024	36.77%	−5.39%
Quadratic	0.014	84.87%	62.18%

**Table 4:** ANOVA table for the quadratic model of MRR.

Source	Full Quadratic			Reduced Quadratic		
	Sum of Squares	F Value	Prob > F	Sum of Squares	F Value	Prob > F
Model	0.0070	3.7404	0.0612	0.0069	5.6642	0.0130
N	0.0008	3.6799	0.1035	0.0012	6.9765	0.0297
f	0.0000	0.0149	0.9067	0.0004	2.4203	0.1584
d	0.0001	0.6400	0.4542	0.0000	0.1592	0.7003
Nf	0.0005	2.6404	0.1553	0.0004	2.4493	0.1562
Nd	0.0001	0.6587	0.4480	-	-	-
fd	0.0018	8.4281	0.0272	0.0023	13.5467	0.0062
N^2^	0.0020	9.4189	0.0220	0.0021	11.9810	0.0086
f^2^	0.0021	9.8556	0.0201	0.0022	12.8268	0.0072
d^2^	0.0000	0.0093	0.9262	-	-	-
Residual	0.0012			0.0014		
Cor Total	0.0083			0.0083		
Standard Deviation	0.0140			0.0132		
Mean	0.2200			0.2232		
Coefficient of Variation%	6.4700			5.90		
R^2^	84.87%			83.21%		
Adjusted R^2^	62.18%			68.52%		

**Table 5:** Optimum parameter reported by CHP and confirmation experimentation.

N (rpm)	f (mm/rev)	d (mm)	Predicted MRR (g/s)	Experiment MRR (g/s)
95	0.2	1.2	0.318	0.326
